# Niagara and Orleans County Veterinary Medical Society

**Published:** 1901-09

**Authors:** Anderson Crowforth

**Affiliations:** Secretary


Secretary.

### NIAGARA AND ORLEANS COUNTY VETERINARY MEDICAL SOCIETY.

THE annual meeting of the Niagara and Orleans County Veterinary Medical Society was held at the City of Lockport, N. Y., August 16th, in the Court House. The President, Dr. Stocking, in a few well-chosen remarks, called the meeting to order, which was the largest and most interesting in the history of the Society. The Prosecuting Committee reported having given Mr. Perry, of Pekin, notice to comply with the law in relation to practising; said Perry agreed to remove his sign and refrain from further practice. Several other cases of the same nature were reported and turned over to the committe with power. The Secretary reported Mr. Mattison, of Niagara Falls, N. Y., as having settled the penalty recovered against him for illegal practising. The Treasurer's report showed that the Society was in a flourishing condition financially, with the balance on the right side of the ledger.

Dr. W. E. Stocking read a paper on "The Veterinarian and Profession."

He said: It is not my intention in this paper to bring before you a subject which enters into the scientific portion of our profession, but to suggest a few ideas along lines which might be well worth our discussion and thought. There are many things in our profession which need our attention and thought aside from the study of disease and its treatment. The small happenings of everyday life are largely responsible for our success. Our profession has long and is still laboring under a cloud of difficulties, for which we are not wholly responsible, but we are struggling to raise this cloud and stand in clear air on equal footing with other professions. The veterinarian until recently was little known to people in general, this man being known to them as "hoss doctor," their interpretation of this term being an uneducated, unintelligent, and usually a morally degraded man, clad about the same as a tramp, and, while going about administering to the poor dumb animals, he never forgets to supply himself with the proper stimulant, of which such men are usually very fond.

The veterinarian, as we understand it, is quite a different personage; he is an intelligent, educated gentleman, representing a profession as noble as any on the face of the earth. Is there one more noble than to relieve the suffering of the domestic animals and protect the lives of our people by guarding their food products? Now, gentlemen, it becomes our duty to demonstrate to the public the difference existing between the educated veterinarian and the quack or uneducated "hoss doctor." It behooves us to take advantage of all things tending to elevate our profession. It is expected by the public that a professional man shall maintain a degree of dignity, and the higher we can elevate and maintain this degree the more our profession is respected by the public. In meeting with the public, to whom we are strangers, we are not first judged by our professional ability, but by our general appearance, of which we may mention our deportment, language, and dress. In a professional way we are often thrown in contact with people whose companionship does not tend to elevate us in the mind of the general public. The less we can have to do with such people, aside from treating them kindly and gentlemanly, the better we are thought of. Our language should be guarded, as profanity detracts from professional elevation. Then comes the last but not least. Of course, our professional duties are a hinderance to our maintaining a high degree of cleanliness, but there is no excuse for our appearing on the street clad in the usual garb of the ancient "hoss doctor" or swipe. I do not mean by this extravagance in dress, but neatness and tidiness about ourselves and our surroundings.

Having reviewed the conduct of the veterinarian, I wish now to bring up for discussion a few points regarding the management of his business. The same rules apply. We are first judged by our appearance rather than our skill. Our instruments and tools used about our business should be kept clean and in good condition; nothing is more unsightly than to see a professional man start to operate with rusty, unwell-kept tools, no matter how trivial the operation. Now comes the putting up of medicines. Our bottles should be of uniform size and shape, with a well-written label pasted upon them and wrapped in a common unprinted paper. All these little things add materially to the significance of the remedy in the eye of our customers. There are many other things along this line I might bring up for your discussion, but I fear I have taken sufficient of your valuable time, as I know there are other papers of great interest to follow.

Dr. M. D. Williams gave an interesting and valuable paper on the treatment of "Milk Fever of Parturition," and Dr. J. O. Moore read one on "Open Joint." All the papers were well received, and drew forth considerable discussion that was of more than ordinary interest.

The following officers were elected for the ensuing year: President, Dr. W. E. Stocking, Medina; Vice-President, Dr. M. D. Williams, Middleport; Secretary-Treasurer, Dr. Anderson Crowforth, Lockport.

For censors: Drs. J. O. Moore, Wilson; J. T. Liddle, West Shelby; G. C. Kelser, Holly; N. Hoffman, Lockport; William R. Hunter, Niagara Falls, N. Y.

Meeting adjourned to meet in Medina in February, 1902.

ANDERSON CROWFORTH,
Secretary.

